# Evaluation of In-House ELISA for Antirabies Antibodies Detection in Domestic Canine

**DOI:** 10.1155/2023/4096258

**Published:** 2023-01-25

**Authors:** Yul Fitria, Niko Febrianto, Rahmi E. Putri, Ibenu Rahmadani, Didik T. Subekti

**Affiliations:** ^1^Bukittinggi Animal Disease Investigation Center, Directorate of Animal Health, Indonesian Ministry of Agriculture, JL. Raya Bukittinggi—Payakumbuh KM 14, Agam 26101, West Sumatra Province, Indonesia; ^2^Center for Biomedical Research, Research Organization for Health, National Research and Innovation Agency, Cibinong Science Center, JL. Raya Jakarta—Bogor Km. 46, Bogor 16911, West Jawa Province, Indonesia; ^3^Indonesian Research Center for Veterinary Science, Agricultural Research and Development Agency, Indonesian Ministry of Agriculture, JL. RE. Martadinata 30, Bogor 16114, West Jawa Province, Indonesia

## Abstract

Indonesia is known to be endemic for rabies in several areas, especially in Sumatra, Kalimantan, Sulawesi, and Flores Islands. Currently, vaccinating dogs has been shown to be the most cost-effective strategy for preventing rabies in humans. Postvaccination monitoring should be carried out to evaluate the success of vaccination by measuring antibody titers in serum of vaccinated dogs. Serological methods for monitoring rabies-specific antibody titers can be carried out using enzyme-linked immunosorbent assay (ELISA) methods as recommended by the World Organization for Animal Health (WOAH). Therefore, the development of the in-house ELISA (BukTi-Vet) that we have carried out in order to support postvaccination monitoring in dogs needs to be evaluated for its diagnostic performance compared to commercial ELISA kits. The diagnostic performance of each ELISA kit was evaluated using 111 known positive and 47 negative serums. Each known positive and negative serum will be tested using the three rabies ELISA kits used in this study. BukTi-Vet is an in-house ELISA for the detection of rabies-specific IgG antibodies that have been developed with sensitivity, specificity, and accuracy of 98.19%, 97.87%, and 98.1%, respectively. Based on the value of its positive and negative clinical utility index, BukTi-Vet is excellent for use in immunoassays directed for confirmatory (0.97) as well as screening (0.94) tests. BukTi-Vet shows a very good agreement with both Platelia II and RFFIT, so it is convincing to be further refined into a diagnostic kit. Tests of field sera from dogs vaccinated with various vaccines should be performed to provide more complete information on diagnostic performance. BukTi-Vet showed a very good agreement with RFFIT, while Pusvetma and Platelia II only showed good agreement. The average value of BukTi-Vet compatibility with RFFIT can reach 94%.

## 1. Introduction

Rabies is one of the oldest zoonotic diseases in the world and causes high fatality in humans. This disease is caused by the Rabies virus (RABV) and other Lyssavirus species from the Rhabdoviridae family [1, 2]. Despite the lack of accurate data on the global burden of neglected tropical diseases, estimates of direct deaths from rabies are most commonly transmitted by bites from rabid animals, especially canines [1]. Most human deaths from rabies occur in Asia and Africa. Estimates of human deaths due to endemic rabies-carrying dogs in Asia and Africa annually exceed 30,000 and 23,000, respectively [1].

Indonesia is known to be endemic for rabies in several areas, especially in Sumatra, Kalimantan, Sulawesi, and Flores Islands [3]. However, outbreaks of rabies in animals and humans have been reported on two islands previously known as rabies-free zones, namely, Bali and Sumbawa Islands. The rabies outbreak in Bali began in 2008–2011 [4–6] and cases continued until 2016 [7]. Recently, a rabies outbreak on the island of Sumbawa occurred in 2018-2019 [8].

Currently, rabies-infected dogs are the major reasons for the high incidence of human rabies, and therefore, vaccinating dogs has shown to be the most cost-effective strategy for preventing rabies in humans [2]. Postvaccination monitoring should be carried out to evaluate the success of vaccination by measuring antibody titers in serum of vaccinated dogs. Serological methods for monitoring rabies-specific antibody titers can be carried out using virus neutralization and enzyme-linked immunosorbent assay (ELISA) methods as recommended by the World Organization for Animal Health (WOAH) previously known as Office International des Epizooties (OIE) [9]. Two well-known virus neutralization methods are the fluorescent antibody virus neutralization (FAVN) and the rapid fluorescent focus inhibition test (RFFIT) [9].

Even though the RFFIT is known to be the most reliable test for the evaluation of successful vaccination, unfortunately, this is time-consuming, expensive, and requires a live rabies virus; sometimes, the results cannot be read off due to the cytotoxic effect on the cells [10, 11]. Virus neutralization technically requires highly skilled technicians, hard to standardize, suffers from some interlaboratory variations, difficult to perform at weekly intervals, and requires containment facilities, which is only conveniently carried out in reference laboratories [10, 12–14]. In order to overcome this, ELISA deserves to be developed as an alternative to RFFIT [11]. ELISA is considered technically simple, affordable, higher in throughput, safer, and rapid compared to RFFIT [1, 15]. ELISA does not require live virus and high-containment facilities, easy to be validated, and has the greater assurance of consistent results [1, 16]. Virus neutralization (RFFIT) and ELISA have also been found to have good compatibility [10, 17]. Therefore, ELISA is very suitable for routine serological testing with a large number of samples, so it is very suitable for monitoring antibody titers after rabies vaccination. The ELISA-based technique was recently adopted by the WOAH or the screening of sera for international pet movement schemes [18]. The commercial ELISA kit commonly used in Indonesia requires quite expensive costs for the imported kit, while domestic products used have low accuracy. Therefore, the development of the in-house ELISA that we have carried out in order to support postvaccination monitoring in dogs needs to be evaluated for its diagnostic performance compared to commercial ELISA kits.

## 2. Materials and Methods

### 2.1. Known Positive, Known Negative, and Positive Control Serum

Animal ethics in this study was not required because all known positive (111 samples) and known negative (47 samples) serum were provided by the Veterinary Pharma Center, Indonesia. The known positive serum is serum from dogs that had been vaccinated with Rabisin® (Boehringer-Ingelheim, Germany) and Rabivet® (Veterinary Pharma Center, Indonesia), while the known negative serum (47 samples) is originally from dogs that had not been vaccinated. The positive control serum was provided by the French Agency for Food, Environmental, and Occupational Health & Safety (ANSES), France. Serum known positives from dogs vaccinated with Rabisin® and Rabivet® were 56 and 55 serum samples, respectively.

### 2.2. Rapid Fluorescent Focus Inhibition Test (RFFIT)

The RFFIT technique was performed by following the procedure described by the WOAH [9]. Briefly, the positive control (0.5 IU/ml) and various dilutions of serum known positive and negative were incubated for 24 h in the presence of the challenge virus standard (CVS) 11 strain suspension infecting baby hamster kidney (BHK)-21 cells. They were grown in Dulbecco's Modified Eagle's Medium (DMEM) (Thermo Scientific, USA) supplemented with 10% fetal bovine serum (FBS) (Thermo Scientific, USA), antibiotic and antifungal using Gibco Antibiotic-Antimycotic (Thermo Scientific, USA), and incubated in an incubator at 37°C with 50% CO_2_ atmosphere. After 24 hours of incubation, the cells were washed and fixed and then incubated with fluorescein isothiocyanate (FITC)-conjugated antirabies monoclonal antibody (Fujirebio, Japan). The serum titers were expressed in IU/ml (international units per milliliter) by determining the last dilution of serum which inhibited 50% of the initial fluorescent foci.

### 2.3. Enzyme-Linked Immunosorbent Assay (ELISA)

The supernatant from cell cultures containing the Pasteur strain rabies virus was centrifuged at 5000 g, 4°C for 15 minutes. The supernatant was then filtered and concentrated using a 10 K protein concentrator (Thermo Scientific, USA). The filtrate was separated, and then, lysis buffer (Qiagen, Germany) was added to it (2 : 1). Protein concentration was determined by the Bradford method using the BioRad Protein Assay (BioRad, France).

The flat bottomed MaxiSorp microplate (Nunc, Denmark) was coated with antigen at a concentration of 2 *μ*g/ml in carbonate-bicarbonate buffer (Sigma Chem., USA) and incubated at 4°C (in a refrigerator) overnight. After three times washing using phosphate-buffered saline containing 0.05% Tween-20 (PBST), it was blocked using 0.5% bovine serum albumin (BSA) (Sigma Chem., USA) in PBST and incubated overnight in the refrigerator. Following three times washing with PBST, the microplate was ready to be used for immunoassay. This kit (including the procedure) hereinafter is referred to as an in-house ELISA rabies kit to be evaluated in this study and coded as BukTi-Vet.

The ELISA procedure using BukTi-Vet will be briefly described in this section. The positive control was diluted to obtain the control serum with titers of 4 IU, 2 IU, 1 IU, 0.5 IU, 0.25 IU, and 0.125 IU. The known positive and known negative serums were diluted at 1 : 200 in PBST, respectively. All serum was put into each well as much as 100 *μ*l. Incubation was carried out at room temperature (23–25°C) for one hour and then followed by three times washing using PBST. A diluted (1 : 20,000) anticanine IgG-HRP conjugate (SouthernBiotech, USA) was added to each well of the microplate. Subsequent incubation and washing were carried out in the same way as the previous step. Finally, the substrate 3,3′, 5,5-tetramethylbenzidine (TMB) solution (Invitrogen, USA) was added and incubated for about 5 minutes before being stopped using 2N H_2_SO_4_. Microplates were read at a wavelength of 450 nm using a BioTek ELx800 microplate reader (BioTek, USA). The absorbance value was then converted into an antibody titer in EU/ml.

The immunoassay procedure using a commercial ELISA kit was carried out according to the protocol described by the manufacturer. The commercial ELISA kit used in this study is a commercial kit that is widely used in Indonesia, namely, the ELISA Rabies® kit (Veterinary Pharma Center, Indonesia) hereinafter referred to as the Pusvetma. Another commercial kit is the Platelia® Rabies II ad usum veterinarium ELISA kit (BioRad, France) and hereinafter referred to as the Platelia II.

### 2.4. Evaluation of ELISA Kit Performance

The diagnostic performance of each ELISA kit was evaluated using 111 known positive and 47 negative serums. Each known positive and negative serum will be tested using the three rabies ELISA kits used in this study. Seropositive and seronegative of the tested serum were determined using a threshold titer value of 0.5 EU/ml. Evaluation of the diagnostic performance of each rabies ELISA kit was analyzed using MedCalc V.20 and the Clinical Utility Index Calculator [19]. Furthermore, an evaluation was also carried out to determine the diagnostic performance of the three rabies ELISA kits against serum derived from the use of different vaccines. The second evaluation was conducted to determine the performance of each rabies ELISA kit against 55 serum known positives from dogs vaccinated with Rabivet® and 47 serum known negatives. The third evaluation was conducted to determine the performance of the three rabies ELISA kits against 56 serum known positives from dogs vaccinated using Rabisin® and 47 serum known negatives.

### 2.5. Interassay Agreement and Simulation of Serial and Parallel Testing

Interassay agreement among the three rabies ELISA kits was carried out on all of 158 known positive and negative serums or also partially on known positive serum from dogs vaccinated with Rabivet® or Rabisin®. The analysis of the results of interassay agreement among the three tests of the rabies ELISA kit was carried out using Agreestat360 [20] and the interpretation of the level of agreement was based on the benchmark from Altman [21]. Serial and parallel test simulations among the three ELISA kits were evaluated to determine the improvement in diagnostic test performance that could be achieved with the combination of the two ELISA kits. Simulation analysis of serial and parallel tests was carried out using Epitools [22].

### 2.6. Interassay Agreement of Three ELISA Kits with RFFIT

The three ELISA kits were also evaluated qualitatively for their interassay agreement compared to the virus neutralization test using the RFFIT method. A total of 48 known positive and negative serums were selected randomly, and the results of their agreement were compared between the three ELISA methods with RFFIT. The test results were analyzed by Agreestat360 [20], and the interpretation of the level of concordance was based on the benchmark from Altman [21].

### 2.7. Evaluation of In-House ELISA Performance by Different Operators

Another series of immunoassays using the in-house ELISA kit (BukTi-Vet) was carried out to evaluate the reliability of the kit by different operators. Operators consist of 2 people who have experience working with ELISA (Operators 1 and 2) and 1 person (Operator 3) who never worked with ELISA at all. Each operator worked on 80 serum known positive and negative which had previously been randomly selected. The results of each operator were analyzed with MedCalc V.20, AgreeStat 360, and Clinical Utility Index Calculator.

## 3. Results

### 3.1. Comparative Performance of In-House ELISA (BukTi-Vet)

Overall, the in-house ELISA kit that has been developed (BukTi-Vet) has excellent accuracy (98.1%) with a sensitivity and specificity of 98.19% and 97.87%, respectively (Table 1). This result was not significantly different (*p* > 0.05) compared to Platelia II which also had an accuracy of 94.94% with a sensitivity and specificity of 94.59% and 95.75%, respectively. On the other hand, the diagnostic performance of BukTi-Vet had a very significant difference (*p* < 0.01) compared to other commercial ELISA kits, namely, Pusvetma which only had an accuracy of 87.34% with a sensitivity and specificity of 82.88% and 97.87%, respectively. However, between the two commercial ELISA kits, namely, Pusvetma and Platelia II, there was no significant difference in diagnostic performance (*p* > 0.05).

**Figure 1:**
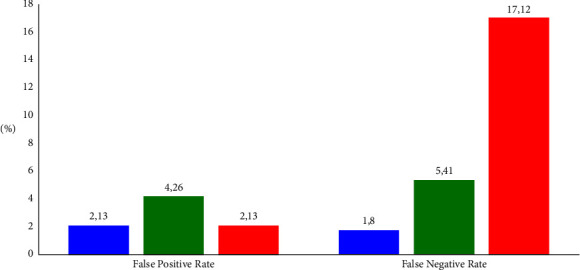
Comparison of the false positive and negative rates of the three ELISA kits in detecting rabies-specific IgG in dog serum. Blue bar, BukTi-Vet; green bar, Platelia II; red bar, Pusvetma.

The clinical utility index (CUI) value consists of a confirmatory test (CUI +ve) and a screening test (CUI −ve). Both clinical utility index values for BukTi-Vet are categorized as an excellent utility. Meanwhile, Pusvetma has a good clinical utility index value for the confirmatory test (CUI +ve) and screening test (CUI −ve). Platelia II has an excellent clinical utility index value for confirmatory tests (CUI +ve) and a good utility index for screening tests (CUI −ve). However, the diagnostic performance of Platelia II is better than Pusvetma because it has a higher clinical utility index score. This result is supported by evidence of a high false-negative rate in Pusvetma (Figure 1).

Evaluation of the diagnostic performance of each known positive serum from two different vaccines showed surprising results. The diagnostic performance of the three ELISA kits against dog serum vaccinated using Rabivet® showed excellent accuracy with values ranging from 92.38% to 97.14% (Table 2). All three showed a diagnostic performance that was not significantly different (*p* > 0.05) based on the area under the curve (AUC) value from the receiver operating characteristic (ROC) curve analysis. However, in the assay using known positive serum from dogs vaccinated with Rabisin®, only Platelia II and BukTi-Vet had excellent accuracy of 96.08% and 97.09% (Table 3). Based on AUC value from the ROC curve analysis, the diagnostic performance of BukTi-Vet and Platelia II showed no difference (*p* > 0.05) when used in known positive serum from dogs vaccinated with Rabisin®. On the other hand, Pusvetma actually showed a significantly different performance (*p* < 0.05) with Platelia II and BukTi-Vet in this assay.

**Figure 2:**
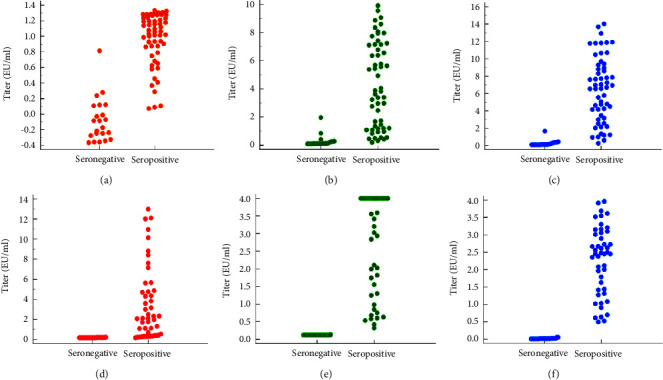
Distribution of IgG antibody titers in known positive serum from dogs vaccinated using Rabivet® (a)–(c) and Rabisin® (d)–(f). Blue dot, BukTi-Vet; green dot, Platelia II; red dot, Pusvetma.

The distribution of IgG antibody titers in known positive serum from dogs vaccinated using Rabivet® and Rabisin® showed a different distribution pattern (Figure 2). The known positive serum from dogs vaccinated with Rabivet® showed an inverse distribution of IgG antibody titers between Pusvetma versus Platelia II and BukTi-Vet. A similar event occurred in the testing of known positive serum from dogs vaccinated with Rabisin®. Measurement of IgG antibody titers in known positive serum from dogs vaccinated with Rabivet® using Platelia II and BukTi-Vet achieved high values (>4 EU/ml), while titer measurements using Pusvetma were only <2 EU/ml (Figures 2(a)–2(c)). In contrast, measurement of IgG antibody titers in known positive serum from dogs vaccinated with Rabisin® using Pusvetma was able to reach values >4 EU/ml, whereas using Platelia II and BukTi-Vet, values were ≤4 EU/ml (Figures 2(d)–2(f)).

### 3.2. Interassay Agreement among Three ELISA Kits and RFFIT

Overall, the results of the analysis of the interassay agreement between BukTi-Vet and Platelia II showed a very good agreement of all coefficient values in accordance with the benchmarking of Altman [21]. Meanwhile, Pusvetma only has a good agreement with Platelia II and BukTi-Vet on all coefficient values (Table 4). The interassay agreement between the three ELISA kits for known negative and positive serum from dogs vaccinated with Rabivet® showed a very good agreement (Table 5). On the other hand, a very good agreement when evaluating known negative and positive serum from dogs vaccinated with Rabisin® only occurred between Platelia II and BukTi-Vet (Table 6).

The analysis of the interassay agreement between two commercial ELISA kits and one in-house ELISA kit showed satisfactory results. BukTi-Vet showed a very good agreement with RFFIT, while Pusvetma and Platelia II only showed good agreement (Table 7). The average value of BukTi-Vet compatibility with RFFIT can reach 94%.

### 3.3. Sensitivity and Specificity Improvement by Serial and Parallel Testing Strategy

Applications in combination with two different ELISA kits sometimes improve diagnostic performance. The combination of two of the three ELISA kits in this study, either serially or in parallel, has been shown to improve the sensitivity and specificity (Table 8). The serial combination of two of the three kits will increase the specificity up to 99%. The combination of two of the three kits in parallel will also increase the sensitivity up to 99%.

### 3.4. Diagnostic Performance of In-House ELISA under Different Operators

In general, BukTi-Vet maintained its diagnostic performance even though it was performed by an operator who had never previously worked with ELISA. Comparing the test results from the three operators, there was no significant difference (*p* > 0.05) in the performance of the BukTi-Vet kit, even though the inexperienced operator (operator 3) caused a decrease in the specificity of 13.04% (Table 9). However, when compared with the original performance of BukTi-Vet, the test results of the third operator caused a very significant reduction in performance (*p* < 0.01), while the test results of the first and second operators did not cause a significant change in their diagnostic performance (*p* > 0.05).

## 4. Discussion

The results of this study proved that Pusvetma has a diagnostic accuracy similar to the previous report of Dartini et al. [23]. They reported that the sensitivity and specificity were 96.8% and 73.5%, respectively, with an estimated diagnostic accuracy of 86.6%. In our study, the sensitivity, specificity, and diagnostic accuracy of Pusvetma were 82.88%, 97.87%, and 87.34% (Table 1). These results also indicate a possible inconsistency of diagnostic performance due to the inverse changes in sensitivity and specificity values between their study and ours. The CUI +ve and CUI −ve values from Pusvetma can be estimated at 0.79 (good utility) and 0.69 (good utility) [23]. Based on this study, it is known that the CUI +ve and CUI −ve values that have been obtained are 0.82 (excellent utility) and 0.69 (good utility). Therefore, the clinical utility of Pusvetma for case finding (confirmation) and screening (ruling out) is categorized as a good utility. However, due to indications of inconsistent diagnostic performance, the ELISA kit (Pusvetma) has the potential to cause doubt and uncertainty to estimate the actual condition, especially in serum samples from animals whose vaccination status and postvaccination immune response are unknown.

Several existing reports on the diagnostic performance of Platelia II are relatively consistent which refers to the sensitivity value being always lower than the specificity. In general, the sensitivity of Platelia II is often below 90%. It has been reported that the sensitivity, specificity, and accuracy of Platelia II were 88.8%, 98.9%, and 90.5%, respectively, when using positive control serum from the WOAH [17]. This diagnostic performance is then used as the final performance in the leaflet that comes with this ELISA kit, namely, Platelia Rabies II ad usum veterinarium (or simply called Platelia II). However, in the same report, the authors also informed that the sensitivity, specificity, and accuracy of Platelia II were 91.1%, 97.8%, and 92.2%, respectively, when using R4b as a positive control serum derived from the kit itself [17]. Another report documented that the sensitivity and specificity of Platelia II were 83.9% and 100% with an estimated accuracy of about 85.3% when using R4b as the positive control serum included in the kit [24]. A similar report also stated that the sensitivity and specificity of the Platelia II ELISA kit were 78.2% and 100% with an estimated accuracy of about 89.1% using the R4b-positive control serum included in the kit [14, 25]. Based on the data reported by Servat et al. [17], the CUI +ve and CUI −ve values were calculated as 0.88 (excellent utility) and 0.62 (fair utility). Otherwise, based on the data reported by Knoop et al. [24], it can be calculated that the value of CUI +ve and CUI −ve is 0.84 (excellent utility) and 0.38 (poor utility). Therefore, the clinical utility of Platelia II for case finding (confirmation) is an excellent utility, while for screening (ruling out), it is a poor or fair utility. Referring to the data from the report above, Platelia II is not suitable for testing directed for screening purposes.

All of the research results that have been reported by several authors above are still below the diagnostic performance of Platelia II as evaluated by this study. Our study showed that the sensitivity, specificity, and diagnostic accuracy of Platelia II were 94.59%, 95.75%, and 94.4%, respectively. The CUI +ve and CUI −ve values in our study were 0.93 (excellent utility) and 0.85 (excellent utility). Therefore, based on our study, the clinical utility of the Platelia II ELISA kit for case finding (confirmation) and screening (ruling out) is categorized as excellent. The clinical utility performance that is close to our study is the report that came from Servat et al. [17] when using R4b as a positive control serum that obtained CUI +ve and CUI −ve values of 0.91 (excellent utility) and 0.66 (good utility). Unfortunately, these data are not referred to as the final diagnostic performance in Platelia II.

BukTi-Vet has provided satisfactory diagnostic performance compared to the other two commercial ELISA kits. The results of the evaluation of diagnostic performance are also consistent, which refers to the sensitivity value being always higher than the specificity, both of which always have a value above 95%. These advantages can be observed from four points. First, the diagnostic accuracy is always above 90%, while the comparison commercial kit is below 90%. Second, the false-negative values were lower than the two comparison ELISA kits (Figure 1). Third, it has very good compatibility with RFFIT compared to the other two ELISA kits (Table 7). Fourth, based on the analysis of the clinical utility index (Table 1), BukTi-Vet is categorized as an excellent utility for confirmation tests (case finding) and screening tests (ruling out). This condition is in accordance with the use of ELISA for the screening of sera for international pet movement schemes as adopted by the WOAH [18]. On the other hand, from this study, it is notable that Platelia II can be categorized as excellent for the confirmation test and quite good for the screening test. Although Pusvetma is categorized as a good utility for confirmation and screening tests, with its inconsistent diagnostic performance, it is necessary to reconsider its use for both purposes.

The correlation of the test between ELISA and RFFIT is often used as the basis for assessing serological diagnostic performance for the detection of rabies-specific antibodies. However, it should be understood that the two methods are actually not always comparable, especially the differences in immunoglobulin classes detected by the two methods [26]. The RFFIT is also known as a functional assay in which the ability of antibodies to inhibit rabies virus infection is determined (known as virus-neutralizing antibodies), while the ELISA is a nonfunctional assay that detects certain classes of binding antibodies to viral antigens [1, 18, 27]. This is one of the main factors that sometimes can lead to incompatibility between the two methods. Under experimental or laboratory conditions, both ELISA and RFFIT techniques have good compatibility, but in field conditions, they are very likely to give different results, so their compatibility is low [18]. Testing between ELISA and RFFIT using controlled laboratory serum (known as positive and negative) can result in very good agreement. This condition is enough to give an expectation that when the ELISA kit is applied to field samples, the difference with RFFIT is not excessive.

Considering this evidence, the differences between Pusvetma and BukTi-Vet in the level of concordance against RFFIT were not associated with differences in the immunoglobulin classes being detected. The basic concept for the development of BukTi-Vet is very similar to that of Pusvetma. Both use native protein from whole virus lysate from the Pasteur strain. These differences should be related to the formulas and techniques we have used for our in-house ELISA development. In simple terms, BukTi-Vet has the advantage of distinguishing seropositive and seronegative compared to Pusvetma giving it a better compatibility with RFFIT.

Evaluation of BukTi-Vet showed fairly good stability of diagnostic performance. This evidence comes from the results of tests carried out by three operators with different skills who were still able to maintain diagnostic performance without significant differences (*p* > 0.05) between the three of them. Despite this, some values of the diagnostic performance indicators were seen to decrease, but the decrease was still within the tolerance threshold so that the overall diagnostic accuracy could still be maintained at a value of 95% (Table 9). The significant decrease compared to the original performance (*p* < 0.05) occurred in the tests carried out by inexperienced operators who had never even worked with ELISA. This also gives an indication that the skill and experience of the operator will affect the performance of the ELISA diagnostic kit used in the real environment.

The interassay agreement between the three ELISA kits showed that BukTi-Vet and Platelia II consistently had a very good agreement (Table 4). The interassay agreement between Pusvetma and Platelia II or BukTi-Vet is only categorized as a good agreement. However, tests involving known positive serum from dogs vaccinated with Rabivet® showed a very good agreement between the three ELISA kits (Table 5). On the other hand, the concordance between tests using known positive serum from dogs vaccinated with Rabisin® only showed a very good agreement between Platelia II and BukTi-Vet. The concordance between Pusvetma with Platelia II or BukTi-Vet on known positive serum from dogs vaccinated with Rabisin® was categorized as good agreement. This indicates that Pusvetma has a tendency of different diagnostic performance in seropositive sera from vaccinated animals using different vaccines.

The ability to measure rabies-specific antibodies from Pusvetma has a better preference for serum from dogs vaccinated with Rabivet®. This is understandable because both the Rabivet® vaccine and the rabies ELISA kit from Pusvetma are produced by the same company. On the other hand, Platelia II and BukTi-Vet have similar detection capabilities against seropositive serum from dogs vaccinated with Rabivet® and Rabisin®. This condition is consistent with the similar distribution of antibody titers from the two vaccine types used in known positive serum, both tested with Platelia II and BukTi-Vet (Figure 2). The distribution of antibody titers tested using Pusvetma on both known positive sera from different vaccine types was in contrast to the results of the other two ELISA kits. These data raise concerns that the diagnostic performance of Pusvetma is not good enough and inconsistent when used to monitor postvaccination antibody levels in serum from dogs using different vaccines. Based on the AUC score of ROC, the diagnostic performance of Pusvetma was not significantly different (*p* > 0.05) from Platelia II and BukTi-Vet , when used for known positive serum from dogs, vaccinated with Rabivet® (Table 2). However, when used for known positive serum from dogs vaccinated with Rabisin® (Table 3), the diagnostic performance of Pusvetma showed a significant difference (*p* < 0.05) with PlateliaII and BukTi-Vet.

The strategy that can be applied to increase the diagnostic sensitivity and specificity of Pusvetma is to perform a combination of serial and parallel testing. The combination of serial testing with Platelia II and BukTi-Vet is calculated to increase the diagnostic specificity up to 99.91% and 99.95% (Table 8). Likewise, parallel testing between Pusvetma and Platelia II and BukTi-Vet resulted in a diagnostic sensitivity of up to 99.07% and 99.69%, respectively. However, the decision to carry out serial and parallel testing is practically not easy and must be done with caution because it remains open to the incompatibility of the test results. The most appropriate action is to use an ELISA kit that has good diagnostic performance and has good test compatibility with other commercial kits.

## 5. Conclusions

BukTi-Vet is an in-house ELISA for the detection of rabies-specific IgG antibodies that have been developed with sensitivity, specificity, and accuracy of 98.19%, 97.87%, and 98.1%, respectively. Based on the value of its clinical utility index, BukTi-Vet is excellent for use in immunoassays directed for confirmatory as well as screening tests. BukTi-Vet shows a very good agreement with both Platelia II and RFFIT, so it is convincing to be further refined into a diagnostic kit. Tests of field sera from dogs vaccinated with various vaccines should be performed to provide more complete information on diagnostic performance.

## Figures and Tables

**Table 1:** Assay performance of three diagnostic kits for rabies-specific IgG detection based on the ELISA technique.

	Pusvetma (%)		Platelia II (%)		BukTi-Vet (%)	
	Mean	95% CI	Mean	95% CI	Mean	95% CI
Sensitivity	82.88	74.57–89.37	94.59	88.61–97.99	98.19	93.64–99.78
Specificity	97.87	88.71–99.95	95.75	85.46–99.48	97.87	88.71–99.95
Accuracy	87.34	81.13–92.09	94.94	90.27–97.79	98.10	94.55–99.61
PPV	98.93	92.96–99.84	98.13	93.11–99.51	99.09	94.00–99.87
NPV	70.77	61.60–78.51	88.24	77.46–94.24	95.83	85.34–98.91
LR+^*∗*^	38.96	5.59–271.32	22.23	5.72–86.34	46.15	6.64–320.91
LR−^*∗*^	0.18	0.12–0.26	0.06	0.03–0.12	0.02	0.01–0.07
AUC^*∗*^	0.90^a^	0.85–0.95	0.95^a,b^	0.91–0.98	0.98^b^	0.95–0.996
CUI +ve^*∗*^	0.82	0.75–0.89	0.93	0.88–0.97	0.97	0.95–0.99
CUI −ve^*∗*^	0.69	0.61–0.77	0.85	0.78–0.91	0.94	0.89–0.98

*Note.* All data in the table are expressed in percent except those marked with an asterisk (^*∗*^). PPV, positive predictive value; NPV, negative predictive value; LR, likelihood ratio; AUC, area under curve; CUI, clinical utility index. AUC with different alphabet notations showed a very significant difference (*p* < 0.01). Benchmark of CUI according to Mitchell [19]: excellent utility (≥0.81), good utility (≥0.64), fair utility (≥0.49), poor utility (<0.49), and very poor utility (<0.36).

**Table 2:** Assay performance of three ELISA kits based on known positive serum from dogs vaccinated with Rabivet®.

	Pusvetma (%)		Platelia II (%)		BukTi-Vet (%)	
	Mean	95% CI	Mean	95% CI	Mean	95% CI
Sensitivity	87.72	76.32–94.92	92.86	82.71–98.02	98.25	90.61–99.96
Specificity	97.92	88.93–99.95	95.75	85.46–99.48	95.83	85.75–99.49
Accuracy	92.38	85.54–96.65	94.18	87.75–97.83	97.14	91.88–99.41
PPV	98.04	87.76–99.71	96.29	86.99–99.02	96.55	87.82–99.09
NPV	87.04	77.02–93.08	91.84	81.37–96.67	97.87	86.82–99.69
LR+^*∗*^	42.11	6.04–293.56	21.82	5.61–84.86	23.58	6.07–91.61
LR−^*∗*^	0.13	0.06–0.25	0.08	0.03–0.19	0.02	0.003–0.13
AUC^*∗*^	0.93^a^	0.86–0.97	0.94^a^	0.88–0.98	0.97^a^	0.92–0.99
CUI +ve^*∗*^	0.86	0.78–0.94	0.89	0.82–0.97	0.95	0.89–0.99
CUI −ve^*∗*^	0.85	0.79–0.91	0.88	0.82–0.94	0.94	0.89–0.98

*Note.* All data in the table are expressed in percent except those marked with an asterisk (^*∗*^). PPV, positive predictive value; NPV, negative predictive value; LR, likelihood ratio; AUC, area under curve; CUI, clinical utility index. AUC with the same alphabet notations showed a not significant difference (*p* > 0.05). Benchmark of CUI according to Mitchell [19]: excellent utility (≥0.81), good utility (≥0.64), fair utility (≥0.49), poor utility (<0.49), and very poor utility (<0.36).

**Table 3:** Assay performance of three ELISA kits based on known positive serum from dogs vaccinated with Rabisin®.

	Pusvetma (%)		Platelia II (%)		BukTi-Vet (%)	
	Mean	95% CI	Mean	95% CI	Mean	95% CI
Sensitivity	78.18	64.99–88.19	96.36	87.47–99.56	98.18	90.28–99.95
Specificity	97.92	88.93–99.95	95.75	85.46–99.48	95.83	85.75–99.49
Accuracy	87.38	79.38–93.11	96.08	90.26–98.92	97.09	91.72–99.39
PPV	97.73	86.02–99.67	96.36	87.21–99.04	96.43	87.42–99.06
NPV	79.66	70.33–86.61	95.75	85.22–98.87	97.87	86.83–99.69
LR+^*∗*^	37.53	5.37–262.32	22.65	5.83–87.98	23.56	6.07–91.55
LR−^*∗*^	0.22	0.14–0.37	0.04	0.01–0.15	0.02	0.003–0.13
AUC^*∗*^	0.88^a^	0.80–0.94	0.96^b^	0.90–0.99	0.97^b^	0.92–0.99
CUI +ve^*∗*^	0.76	0.65–0.88	0.93	0.87–0.99	0.95	0.89–0.99
CUI −ve^*∗*^	0.78	0.71–0.85	0.92	0.87–0.97	0.94	0.89–0.98

*Note.* All data in the table are expressed in percent except those marked with an asterisk (^*∗*^). PPV, positive predictive value; NPV, negative predictive value; LR, likelihood ratio; AUC, area under curve; CUI, clinical utility index. AUC with different alphabet notations showed a significant difference (*p* < 0.05). Benchmark of CUI according to Mitchell [19]: excellent utility (≥0.81), good utility (≥0.64), fair utility (≥0.49), poor utility (<0.49), and very poor utility (<0.36).

**Table 4:** Interassay agreement among three ELISA methods in detecting rabies-specific IgG.

Methods	Pusvetma vs. Platelia II		Pusvetma vs. BukTi-Vet		BukTi-Vet vs. Platelia II	
	Coeff.	95% CI	Coeff.	95% CI	Coeff.	95% CI
Cohen's kappa	0.70	0.59–0.82	0.74	0.63–0.85	0.87	0.78–0.95
Scott's pi	0.70	0.58–0.82	0.74	0.63–0.85	0.87	0.78–0.9
Krippendorff's alpha	0.70	0.59–0.82	0.74	0.63–0.85	0.87	0.78–0.9
Gwet's AC_1_	0.74	0.64–0.85	0.78	0.68–0.88	0.90	0.84–0.97
Brennan–Prediger	0.72	0.61–0.83	0.76	0.66–0.86	0.89	0.81–0.96
Percent agreement	0.86	0.81–0.92	0.88	0.83–0.93	0.94	0.91–0.98

*Note.* Altman benchmark for agreement coefficients [21]: very good (0.8–1), good (0.6–0.8), moderate (0.4–0.6), fair (0.2–0.4), and poor (<0.2).

**Table 5:** Interassay agreement among three ELISA kits based on known positive serum from dogs vaccinated with Rabivet®.

Methods	Pusvetma vs. Platelia II		Pusvetma vs. BukTi-Vet		BukTi-Vet vs. Platelia II	
	Coeff.	95% CI	Coeff.	95% CI	Coeff.	95% CI
Cohen's kappa	0.81	0.69–0.92	0.87	0.77–0.96	0.88	0.79–0.98
Scott's pi	0.81	0.69–0.92	0.87	0.77–0.96	0.88	0.79–0.98
Krippendorff's alpha	0.81	0.69–0.92	0.87	0.77–0.96	0.88	0.79–0.98
Gwet's AC_1_	0.81	0.69–0.92	0.87	0.77–0.96	0.88	0.79–0.98
Brennan–Prediger	0.81	0.69–0.92	0.87	0.77–0.96	0.88	0.79–0.98
Percent agreement	0.90	0.85–0.96	0.93	0.89–0.98	0.94	0.89–0.99

*Note.* Altman benchmark for agreement coefficients [21]: very good (0.8–1), good (0.6–0.8), moderate (0.4–0.6), fair (0.2–0.4), and poor (<0.2).

**Table 6:** Interassay agreement among three ELISA kits based on known positive serum from dogs vaccinated with Rabisin®.

Methods	Pusvetma vs. Platelia II		Pusvetma vs. BukTi-Vet		BukTi-Vet vs. Platelia II	
	Coeff.	95% CI	Coeff.	95% CI	Coeff.	95% CI
Cohen's kappa	0.77	0.65–0.89	0.75	0.63–0.88	0.94	0.88–1
Scott's pi	0.77	0.65–0.89	0.75	0.63–0.88	0.94	0.88–1
Krippendorff's alpha	0.77	0.65–0.90	0.75	0.63–0.88	0.94	0.88–1
Gwet's AC_1_	0.77	0.65–0.90	0.75	0.63–0.88	0.94	0.88–1
Brennan–Prediger	0.77	0.65–0.90	0.75	0.63–0.88	0.94	0.88–1
Percent agreement	0.89	0.82–0.95	0.88	0.81–0.94	0.97	0.94–1

*Note.* Altman benchmark for agreement coefficients [21]: very good (0.8–1), good (0.6–0.8), moderate (0.4–0.6), fair (0.2–0.4), and poor (<0.2).

**Table 7:** Interassay agreement among three ELISA kits with RFFIT.

Methods	Pusvetma vs. RFFIT		Platelia II vs. RFFIT		BukTi-Vet vs. RFFIT	
	Coeff.	95% C.I	Coeff.	95% CI	Coeff.	95% CI
Cohen's kappa	0.64	0.35–0.93	0.72	0.49–0.95	0.80	0.58–1
Scott's pi	0.64	0.35–0.94	0.71	0.47–0.95	0.80	0.58–1
Krippendorff's alpha	0.64	0.35–0.94	0.72	0.47–0.96	0.80	0.58–1
Gwet's AC_1_	0.84	0.69–0.99	0.83	0.68–0.99	0.91	0.79–1
Brennan–Prediger	0.78	0.59–0.97	0.79	0.61–0.97	0.87	0.73–1
Percent agreement	0.89	0.79–0.98	0.89	0.80–0.90	0.94	0.86–1

*Note.* Altman benchmark for agreement coefficients [21]: very good (0.8–1), good (0.6–0.8), moderate (0.4–0.6), fair (0.2–0.4), and poor (<0.2).

**Table 8:** Estimated diagnostic performance with a combination of serial and parallel tests among the three ELISA kits for rabies detection.

	Pusvetma	Platelia II	Series	Parallel
Sensitivity	82.88%	94.59%	78.40%	99.07%
Specificity	97.87%	95.75%	99.91%	93.71%

	Pusvetma	BukTi-Vet	Series	Parallel
Sensitivity	82.88%	98.19%	81.38%	99.69%
Specificity	97.87%	97.87%	99.95%	95.79%

	BukTi-Vet	Platelia II	Series	Parallel
Sensitivity	98.19%	94.59%	92.88%	99.90%
Specificity	97.87%	95.75%	99.91%	93.71%

**Table 9:** Assay performance of in-house ELISA BukTi-Vet under different skill operators.

	Original (%)^§^		Operator 1 (%)		Operator 2 (%)		Operator 3 (%)	
	Mean	95% CI	Mean	95% CI	Mean	95% CI	Mean	95% CI
Sensitivity	98.19	93.64–99.78	98.25	90.61–99.96	96.49	87.89–99.57	98.25	90.61–99.96
Specificity	97.87	88.71–99.95	91.30	71.96–98.93	91.30	71.96–98.93	78.26	56.29–92.54
Accuracy	98.10	94.55–99.61	96.25	89.43–99.22	95.00	87.69–98.62	92.50	84.39–97.19
PPV	99.09	94.00–99.87	96.55	88.16–99.06	96.49	87.96–99.04	91.80	83.75–96.05
NPV	95.83	85.34–98.91	95.46	74.98–99.33	91.30	72.79–97.63	94.74	71.82–99.22
LR+^*∗*^	46.15	6.64–320.91	11.29	3.00–42.49	11.09	2.95–41.76	4.52	2.08–9.82
LR−^*∗*^	0.02	0.01–0.07	0.02	0.003–0.14	0.04	0.01–0.15	0.02	0.003–0.16
AUC^*∗*^	0.98^a^	0.95–0.99	0.95^a,b^	0.87–0.99	0.94^a,b^	0.86–0.98	0.88^b^	0.79–0.94
CUI +ve^*∗*^	0.97	0.95–0.99	0.95	0.89–0.99	0.93	0.87–0.99	0.90	0.83–0.97
CUI −ve^*∗*^	0.94	0.89–0.98	0.87	0.78–0.96	0.83	0.73–0.93	0.74	0.61–0.87

*Note.* ^§^Original diagnostic performance as described in Table 1. All data in the table are expressed in percent except those marked with an asterisk (^*∗*^). PPV, positive predictive value; NPV, negative predictive value; LR, likelihood ratio; AUC, area under curve; CUI, clinical utility index. AUC with the same notations showed a not significant difference (*p* > 0.05). Benchmark of CUI according to Mitchell [19]: excellent utility (≥0.81), good utility (≥0.64), fair utility (≥0.49), poor utility (<0.49), and very poor utility (<0.36).

## Data Availability

The data used to support the findings of this study are available from the corresponding author upon request.
